# Utilization of physical therapy, speech therapy and occupational therapy by children and adolescents in Germany. Results of the cross-sectional KiGGS Wave 2 study and trends

**DOI:** 10.17886/RKI-GBE-2018-097

**Published:** 2018-12-12

**Authors:** Alexander Rommel, Birte Hintzpeter, Dominika Urbanski

**Affiliations:** Robert Koch Institute, Berlin, Department of Epidemiology and Health Monitoring

**Keywords:** PHYSICAL THERAPY, SPEECH THERAPY, OCCUPATIONAL THERAPY, HEALTH MONITORING, KIGGS

## Abstract

Allied health services such as physical therapy, speech therapy and occupational therapy contribute to the early treatment of health disorders in children and adolescents and promote a healthy development. This article describes the utilization of these allied health services by children and adolescents in Germany and analyses its association with demographic and social factors. The analyses are based on the second wave of the German Health Interview and Examination Survey for Children and Adolescents (KiGGS Wave 2, 2014-2017) including 15,023 participants. Trends are calculated in comparison with the KiGGS baseline study (2003-2006). Within one year, 9.6% of children and adolescents in Germany use physical therapy, 6.1% speech therapy and 4.0% occupational therapy. Speech therapy and occupational therapy are used more frequently by boys than by girls. The utilization of speech therapy is highest among 3- to 6-year-olds with 15.0%. Occupational therapy (8.3%) is most frequently used by 7- to 10-year-olds and physical therapy (16.9%) by 14- to 17-year-olds. Social differences are evident mainly in the higher utilization of occupational therapy and speech therapy and a lower utilization of physical therapy by socially disadvantaged children and adolescents. Over the last ten years, the utilization of speech therapy and physical therapy in children and adolescents has increased significantly.

## 1. Introduction

In the coming years, the health care system will face major challenges, which will not only lead to a quantitative expansion of the overall demand for care, but also to qualitatively changed requirements. The allied health services, too, are challenged by an increase in the complexity of care regarding counselling, patient education and therapeutic practice [1]. In the present article, allied health services are mainly understood to be occupational therapy, speech therapy and physical therapy financed by statutory health insurances. In 2016, statutory health insurers in Germany spent EUR 6.5 billion or 2.9% of their budget on these services, a figure which continues to rise [2]. Unlike other specialist services provided at an older age, allied health services already play a key role in the provision of healthcare to children and adolescents [2-6].

**KiGGS Wave 2**

Second follow-up to the German Health Interview and Examination Survey for Children and Adolescents

**Data owner:** Robert Koch Institute

**Aim:** Providing reliable information on health status, health-related behaviour, living conditions, protective and risk factors, and health care among children, adolescents and young adults living in Germany, with the possibility of trend and longitudinal analyses

**Study design**: Combined cross-sectional and cohort study

**Cross-sectional study in KiGGS Wave 2**

**Age range:** 0-17 years

**Population:** Children and adolescents with permanent residence in Germany

**Sampling:** Samples from official residency registries - randomly selected children and adolescents from the 167 cities and municipalities covered by the KiGGS baseline study

**Sample size:** 15,023 participants

**KiGGS cohort study in KiGGS Wave 2**

**Age range:** 10-31 years

**Sampling:** Re-invitation of everyone who took part in the KiGGS baseline study and who was willing to participate in a follow-up

**Sample size:** 10,853 participants

**KiGGS survey waves**

► KiGGS baseline study (2003-2006), examination and interview survey
► KiGGS Wave 1 (2009-2012), interview survey
► KiGGS Wave 2 (2014-2017), examination and interview survey

More information is available at www.kiggs-studie.de/english

Occupational therapy supports and accompanies people whose ability to act is limited or threatened due to limited motor skills, sense disorders as well as mental disorders [7]. Limitations in motor skills, as well as mental health problems are typical indications for occupational therapy among children and adolescents. Therapy includes measures to improve gross and fine motor skills, but also behaviour therapy approaches, concentration exercises or the development of social competence. Speech therapy is the specialist discipline that deals with language, speech, voice and swallowing impairments [7]. Among children and adolescents, disorders of fluency of speech (such as stammering) or of speech control (such as having a lisp) are frequent medical indications. Treatment options include exercises to improve articulation, breathing and vocalisation, as well as speech fluency, finding words and grammar exercises. Physical therapy, through specific training and movement exercises, external treatments (such as heat therapy treatments) or manual therapy, aims to restore, improve or maintain the body’s capacity for movement and other functions [7]. For children and adolescents, medical indications for treatment mostly concern disorders of the musculoskeletal system and connective tissue, for example malpositionings or scoliosis (spinal curvature). Back pain is gaining importance as an indication for treatment in adolescents [2, 4].

Physical therapy, speech therapy and occupational therapy comprise counselling, diagnostics and treatment and are applied in prevention, acute care and rehabilitation of children and adolescents. Therefore, these therapies are referenced in numerous medical guidelines, for example in the diagnosis and therapy of speech fluency disorders [8], development disorders [9] or non-specific low-back pain [10]. The importance of such allied health services in healthcare provision to children and adolescents is reflected in utilization figures. Results from the baseline study of the German Health Interview and Examination Survey for Children and Adolescents (KiGGS baseline study, 2003-2006) show that 2.4% of 3- to 13-year-olds were given occupational therapy within 12 months. 6.4% of 0- to 17-year-olds used physical therapy services [5, 6]. According to the health insurance provider Allgemeine Ortskrankenkassen (AOK), 9.8% of all insured girls and 13.8% of all insured boys up to the age of 14 years used allied health services such as physical therapy, speech therapy or occupational therapy in 2016. Developmental disorders (Code F80-F89 of the International Statistical Classification of Diseases and Related Health Problems, 10th revision, ICD-10) account for more than half of all prescriptions at this age, plus around 10% of behavioural disorders (ICD-10 F90-F98) [2]. Among the three allied health services discussed here, speech therapeutic measures for insured children and adolescents up to the age of 14 were most frequently paid for by the AOK amounting to around 48%. Occupational therapy accounts for around 27% and physical therapy for around 21% [2]. The same patients often receive different therapies. Adolescents who receive speech therapy for language development disorders for example frequently also use occupational therapy [11].

The role of physical therapy, speech therapy and occupational therapy in the provision of care is also reflected in the health policy debate. Since 2008, section 63 of Book 5 of the German Social Code provides therapists with greater autonomy to decide on specific therapies. Since 2017, with the Gesetz zur Stärkung der Heil- und Hilfsmittelversorgung, statutory health insurers have been committed to develop pilot projects. Doctors can now provide blank prescriptions for physical therapy, speech therapy or occupational therapy, and therapists then decide on the appropriate treatment (section 64d of Book 5 of the German Social Code). The implementation in routine care is already foreseeable [12]. In addition, as early as 2012, the Wissenschaftsrat called for greater academization of healthcare professions and the expansion of healthcare research in the field of allied health services [1]. Similarly, the current report of the Sachverständigenrat zur Begutachtung der Entwicklung im Gesundheitswesen supports a further strengthening of the role of physical therapy in the context of interdisciplinary treatment approaches under the prerequisite of stronger evidence-based and academic approaches [13]. Against this background, it seems important to improve the transparency of healthcare provision in the field of allied health services such as physical therapy, speech therapy and occupational therapy [3, 4, 14].

This article contributes to this discussion by thoroughly analysing the utilization of physical therapy, speech therapy and occupational therapy by children and adolescents in Germany on the basis of the second wave of the German Health Interview and Examination Survey for Children and Adolescents (KiGGS Wave 2, 2014-2017). Beyond showing the frequency of service use, developments over time are considered. The association between utilization of these allied health services and social factors is also analysed. Previous studies have suggested that even when there is a medical indication for treatment, social factors play a role in the utilization of physical therapy, speech therapy and occupational therapy [5, 6, 15]. Therefore the question arises as to whether all social groups benefit equally from these services when they need care.

## 2. Methodology

### 2.1 Sample design and study implementation

KiGGS is part of the health monitoring system at the Robert Koch Institute (RKI) and includes repeated cross-sectional surveys of children and adolescents aged 0 to 17 years that are representative for Germany. The field phase is always carried out over several years. The KiGGS baseline study (2003-2006) was conducted as an examination and interview survey, KiGGS Wave 2 (2014-2017) as a combined examination and interview survey. The concept and design of KiGGS have been described in detail elsewhere [16-19]. Participants were selected randomly from the official registries of the 167 cities and municipalities representative for Germany which had already been chosen for the KiGGS baseline study. A number of activities have been conducted to increase study participation and to improve the sample composition. 15,023 children and adolescents (7,538 girls and 7,485 boys) took part in KiGGS Wave 2 (response 40.1%). Response rates were calculated, based on the Response Rate 2 of the American Association for Public Opinion Research (AAPOR) [20]. 17,641 girls and boys aged 0 to 17 years took part in the KiGGS baseline study, with a response rate of 66.6% [18].

### 2.2 Indicators

Indicators on utilization of allied health services were measured in KiGGS Wave 2 based on the self-reported information of participants (11- to 17-years-old) or their legal guardians (0- to 10-years-old) in a self-administered questionnaire. The question asked was: ‘Which of the following types of therapists has your child/have you seen during the last 12 months and how often?’ For the use of allied health services, the answer categories were physical therapist, speech therapist or occupational therapist. In the KiGGS baseline study, the utilization of occupational therapy was not explicitly asked. As data on this type of therapy was only collected through free text entries, no trends for occupational therapy can be shown.

In addition to age and gender, the socioeconomic status (SES) and the migration background of children and adolescents were taken into account as sociodemographic factors. Family SES was measured through an index based on the information parents provided on educational background, occupational status and equivalised household income. The SES index allows for a differentiation between low, medium and high status groups [21]. The migration background of participants was established in line with the previous KiGGS waves based on a child’s or adolescent’s country of birth, their parents’ country of birth as well as citizenship [22]. A migration background applies if either one or both parents were not born in Germany or do not have German citizenship. If the child itself has immigrated from another country, a migrant background is assigned if at least one parent was not born in Germany.

Individual diseases are less suited as indicators of a medical need that may lead to physical therapy, speech therapy or occupational therapy compared to more comprehensive indicators that were covered by the KiGGS study. Officially recognised disabilities, mental health problems and chronic diseases were taken into account, as well as general health-related limitations and medically treated injuries during the last 12 months. Recognised disabilities were assessed by asking guardians: ‘Has your child got a disability that has been officially recognised by a public institution?’ The indicator of mental health problems is based on the answers provided by parents of 3- to 17-year-old children and adolescents in the Strengths and Difficulties Questionnaire (SDQ). The results were subdivided into three categories of ‘abnormal’, ‘borderline’ and ‘normal’ [23]. Moreover, following the European Health Module (MEHM) [24], the standardised instrument at the EU level, legal guardians were asked: ‘Does your child suffer from one or several long-term, chronic diseases or health issues?’ Data on health limitations was furthermore collected by using the Children with Special Health Care Needs (CSHCN) screener, which was developed to identify children with special care needs [25]. For this purpose, three questions were combined into a dichotomous measure of health-related limitations in everyday life. Finally, data on medically treated injuries were also collected by asking guardians: ‘Has your child suffered an injury (for example due to an accident, poisoning or violence) requiring treatment by a doctor during the last 12 months?’

### 2.3 Statistical methods

The analyses are based on the data from 13,692 participants (6,910 girls and 6,782 boys) aged 0 to 17 years. 1,331 participants were excluded because they did not provide information on utilization of physical therapy, speech therapy or occupational therapies over the last 12 months. The results are presented as prevalence with 95% confidence intervals (95% CI) stratified according to gender, age and SES. The calculations were carried out using a weighting factor that corrected for deviations within the sample from the population structure with regard to regional structure (rural area/urban area), age (in years), gender, federal state (as of 31 December 2015), German citizenship (as of 31 December 2014) and the parents’ level of education based on the Comparative Analysis of Social Mobility in Industrial Nations (CASMIN classification) (Microcensus 2013 [26]).

To analyse trends, we used data on the variable utilization of speech therapy from 31,026 participants (15,411 girls, 15,615 boys) of the KiGGS baseline study. For the variable utilization of physical therapy, data from 31,025 participants (15,410 girls, 15,615 boys) were available. 1,637 participants were excluded because they did not provide information on the utilization of speech therapy. The same was true for 1,638 participants in case of physical therapy. Analyses of trends on the utilization of occupational therapy are not possible since this data was not explicitly assessed in the KiGGS baseline study. To describe trends between the KiGGS baseline study and KiGGS Wave 2, age-standardised prevalences were calculated for the German population as of 31 December 2015. A new weighting factor was applied to the data of the KiGGS baseline study that considers parents’ levels of education and federal state in addition to the factors considered by the original weighting factor in line with KiGGS Wave 1 and KiGGS Wave 2.

To evaluate the association between sociodemographic factors and the utilization of physical therapy, speech therapy and occupational therapy, binary logistic regression was applied. For this purpose, the indicators of medical need – recognised disability, mental health problems, chronic diseases, health-related limitations and medically treated injuries – were used as control variables. Adjusted odds ratios (aOR) with 95% confidence intervals express the factor by which the statistical chance of using these allied health services for a specific group (e.g. low SES) deviates in relation to the respective reference group (e.g. high SES). Depending on the indicator, different numbers of participants had to be excluded from the analysis due to missing data.

All analyses were conducted with Stata 15.1 (Stata Corp., College Station, TX, USA, 2017) using the KiGGS Wave 2 data (Version 09). Survey procedures for complex samples were used in all analyses to adequately account for the clustering of participants in sample points and to consider weighting in the calculation of confidence intervals and p-values [27]. A statistically significant difference between groups is assumed if the p-value was lower than 0.05.

## 3. Results

Overall, 9.6% of 0- to 17-year-old children and adolescents use physical therapy services within one year. At 6.1%, the utilization of speech therapy within the same time frame is slightly lower and for occupational therapy it is 4.0%. Whereas across all age groups no gender differences are evident in the utilization of physical therapy, there are significant differences in the utilization of speech and occupational therapy between girls and boys. With 4.9%, girls use speech therapy significantly less often compared to 7.1% of boys. The same holds true for the utilization of occupational therapy, where the figures are 2.7% of girls and 5.4% of boys. A small proportion of children and adolescents moreover have more than one therapy within one year with higher rates in boys (2.1%) than in girls (1.6%). Occupational and speech therapy in particular are most frequently combined in these cases. Around 18.3% of children and adolescents in speech therapy also use occupational therapy.

While boys use speech and occupational therapy significantly more frequently, the mean number of contact during therapy is slightly lower in boys. Girls in speech therapy on average see their therapist 18.0 times per year, boys 16.7 times. Similarly, girls in occupational therapy have therapy services 23.6 times per year on average, whereas the contact frequency for boys is 18.9 times. The differences for the utilization of physical therapy with 13.9 contacts for girls and 11.6 contacts for boys are slightly smaller (data not shown).

Analysed by age, the utilization of physical therapy, speech therapy and occupational therapy is clearly concentrated in certain age groups. Physical therapy utilization is quite high in the 0- to 2-year age group. The figure for 3- to 7-year-olds is significantly smaller and then rises again with age. The highest utilization rate of physical therapy is found in the 14- to 17-year age group with 16.9%. At this age, 19.3% of girls and 14.4% of boys make use of physical therapy services within one year. Marked gender differences therefore only emerge in the older age groups.

**Figure 1:**
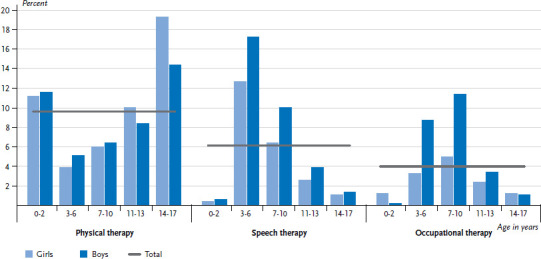
Utilization of physical therapy, speech therapy and occupational therapy in the last 12 months among 0- to 17-year-olds according to gender and age (n=6,910 girls, n=6,782 boys) Source: KiGGS Wave 2 (2014-2017)

Speech and occupational therapy however dominate at pre- and primary school age. Like shown for physical therapy, the differences between girls and boys in speech therapy are particularly apparent in the age group with the highest utilization rates. At 15.0%, the utilization of speech therapy is highest in the 3- to 6-year age group. At this age, 12.7% of girls and 17.2% of boys use speech therapy within one year. With age, this proportion decreases considerably. Whereas the utilization of speech therapy reaches its peak before school entry, the use of occupational therapy peaks after school entry. At 8.3% utilization of occupational therapy was highest in the 7- to 10-year age group. At this age, 5.0% of girls and 11.4% of boys use occupational therapy within a 12-month period (Figure 1 and Table 1).

Socioeconomic status only marginally affects utilization of allied health services. The only exception is the utilization of occupational therapy among girls which is more frequent in low than in medium and high status groups. Similarly, there are no pronounced differences in the utilization of these allied health services between children and adolescents from migrant and non-migrant background families (Table 1).

When looking at the utilization of physical therapy, speech therapy and occupational therapy with regard to the indicators of medical need, there are clear differences in some cases. Most frequently, all three therapies are used by children and adolescents with a officially recognised disability. Health-related limitations also lead to a significantly higher utilization. Mental health problems, chronic diseases and medically treated injuries by contrast show less pronounced associations with the use of allied health services (Table 2). Injuries are primarily associated with the use of physical therapy. Significant differences between the genders regarding the relationship between indicators of medical need and the use of physical therapy, speech therapy and occupational therapy are not apparent (data not shown).

**Figure 2:**
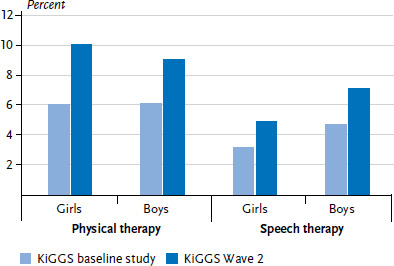
Trends in the utilization of physical therapy and speech therapy in the last 12 months among 0- to 17-year-olds between the KiGGS baseline study (n=8,501 girls, n=8,833 boys) and KiGGS Wave 2 (n=6,910 girls, n=6,782 boys) Source: KiGGS baseline study (2003-2006), KiGGS Wave 2 (2014-2017)

Data on the utilization of physical therapy and speech therapy was collected in a similar manner in the KiGGS baseline study and KiGGS Wave 2 allowing the assessment of trends across these two points in time. Between the survey periods 2003 to 2006 and 2014 to 2017, there was a significant increase in the utilization of physical therapy as well as speech therapy for both genders (Figure 2). Whereas in the KiGGS baseline study, 6.0% of girls and 6.1% of boys used physical therapy services, in KiGGS Wave 2 it was 10.1% of girls and 9.1% of boys. For speech therapy, the increase between the two KiGGS survey Waves was from 3.2% to 4.9% for girls and 4.7% to 7.1% for boys (Figure 2).

Differentiated by sociodemographic factors, a largely uniform increase can be seen for both types of therapy in all age groups as well as according to SES and migration background (data not shown). There is no significant trend, only in the case of speech therapy in the 0- to 2-year age group, as well as among children and adolescents with high SES.

The results of the multivariate analysis show that even when statistically controlled for indicators of medical need, differences according to age, gender, SES and migration background persist (Table 3). Multivariate analysis for example shows that boys use physical therapy significantly less often, but occupational or speech therapy more frequently. Moreover, the utilization of physical therapy over the last 12 months is significantly lower in the younger age groups compared to the 14- to 17-year-old reference group. The results for speech and occupational therapy in contrast show that utilization in all younger age groups is significantly higher than for the 14- to 17-year-olds. Moreover, the multivariate model produces clearer differences by SES than the descriptive analysis. While the utilization of speech and occupational therapy among children and adolescents with low and medium SES is higher than for their peers with high SES, the utilization of physical therapy among children and adolescents with low SES is lower than that of their peers with high SES. In contrast, also when controlling for indicators of medical need, the tendency is consistently towards a lower utilization of all three therapies in children and adolescents with a migration background. The relationship between migration background and the utilization of allied health services, however, is only statistically significant for the utilization of physical therapy.

## 4. Discussion

Physical therapy, speech therapy and occupational therapy are a key area of healthcare and significantly contribute to the healthy development of children and adolescents. 9.6% of children and adolescents in Germany use physical therapy within one year, 6.1% use speech therapy and 4.0% occupational therapy. Across all age groups, boys use speech therapy and occupational therapy more often than girls. In contrast, the utilization of physical therapy is higher for 14- to 17-year-old girls. Certain age groups stand out with particularly high rates. Utilization of speech therapy is particularly frequent at preschool and primary school age and highest for 3- to 6-year-olds with 15.0%. At 8.3%, use of occupational therapy is most frequent at primary school age (7 to 10 years). The utilization of physical therapy shows a peak for the 0- to 2-year age group, then decreases sharply and, after a gradual increase, reaches the highest utilization rate among 14- to 17-year-olds with 16.9%.

Since the KiGGS baseline study, the utilization of the three forms of therapy has increased significantly. This trend can also be found in the analyses of routine data. Prescriptions of physical therapy, speech therapy and occupational therapy have increased. Even if the rates of children and adolescents treated has stagnated in recent years [2], our analyses suggest a long-term increase in the proportion of children and adolescents that use allied health services. Analyses based on routine data also confirm the differences regarding age and gender [2]. In contrast, for the association between socioeconomic status or migration background and the utilization of physical therapy, speech therapy and occupational therapy the findings are more heterogeneous. The higher utilization rates of physical therapy services among the socially better off and people without a migration background has already been found in studies among adults [15]. However, former analyses of the use of occupational therapy and physical therapy in children and adolescents showed no clear associations with socioeconomic characteristics or migration background [5, 6].

In the present analysis, even when controlling for actual medical needs, statistical correlations such as between age and the utilization of physical therapy, speech therapy and occupational therapy by children and adolescents remain. This indicates that medical need has not been fully considered. While there are clear associations between the applied indicators for medical need and utilization of these services, the data from the KiGGS study do not allow analysing for what specific reason the respective therapies were actually carried out. Moreover, the applied indicators fail to sufficiently capture temporary, reversible diseases or disorders.

Routine data of statutory health insurers shows that such indications can explain age specific utilization rates. During the first two years of life, diagnoses related to development disorders of motor functions which lead to the utilization of physical therapy dominate. In the group of 3- to 5-year-olds, diagnoses such as speech disorders requiring speech therapy show a high prevalence. In the group of 6- to 8-year-old children developmental disorders and hyperkinetic disorders such as attention deficit/hyperactivity disorder (ADHD) are common indications that often go hand in hand with occupational therapy [2]. In the case of physical therapy, the data presented clearly shows that the patterns that develop among 14- to 17-year-olds already resemble those found among adults. Analyses based on studies conducted at the Robert Koch Institute consistently confirm a high 12-month prevalence for the utilization of physical therapy by more than a fifth of the adult population with significantly higher rates for women [15, 28]. Among both, adolescents and adults this is increasingly related to the diagnosis of back pain [2, 4].

Ultimately, it is difficult to assess the extent to which an appropriate level of prescriptions exists with both survey and routine data. On the one hand, it is noticeable that in regions where the number of service providers increases, prescriptions also rise [7]. Analyses from the German Health Interview and Examination Survey for Adults (DEGS1) by the Robert Koch Institute suggest a correlation between the density of service providers and the utilization of physical therapy for the adult population [15]. This raises the question as to whether the respective therapies are generally prescribed according to need. On the other hand, for occupational therapy it has been demonstrated that not all children and adolescents with an indication also receive treatment. It is therefore important to ask in which groups of children and adolescents there may be undersupply and how needs-based care can be ensured in these cases [3].

The overall expenditure and dynamics of expenditure in the provision of allied health services such as physical therapy, speech therapy and occupational therapy are disproportionate to the low level of attention paid to this area of care in health research [29]. Even though these therapies are considered as effective treatment approaches with hardly any side effects, health insurers warn that there is a partial lack of evidence-based decision-making aids for healthcare practice [3, 4]. All the more, it is the responsibility of all actors both, to make service provision in the field of allied health services more transparent and to increase knowledge about evidence-based procedures and therapies.

## Key statements

- Every year, 9.6% of 0- to 17-year-olds are treated with physical therapy. Speech therapy (6.1%) and occupational therapy (4.0%) are used less frequently.
- In particular, speech therapy and occupational therapy are used more frequently by boys than by girls.
- The highest level of use is found for speech therapy in 3- to 6-year-olds (15.0%), for occupational therapy in 7- to 10-year-olds (8.3%) and for physical therapy in 14- to 17-year-olds (16.9%).
- Over the last ten years, the utilization of speech therapy and physical therapy among children and adolescents has increased significantly.
- Children and adolescents with a low socioeconomic status are less likely to seek physical therapy, but more likely to seek speech therapy and occupational therapy.

## Figures and Tables

**Table 1:** Utilization of physical therapy, speech therapy and occupational therapy in the last 12 months among 0-to 17-year-olds according to gender, age, socio economic status and migration background (n=6,910 girls, n=6,782 boys) Source: KiGGS Wave 2 (2014-2017)

Physical therapy			Speech therapy		Occupational therapy	
%		(95% Cl)	%	(95% CI)	%	(95% CI)
**Girls (total)**	**10.1**	**(9.1-11.2)**	**4.9**	**(4.3-5.7)**	**2.7**	**(2.2-3.3)**
**Age group**						
0-2 Years	11.2	(8.6-14.5)	0.4	(0.1-1.2)	1.2	(0.4-3.6)
3-6 Years	3.9	(2.7-5.8)	12.7	(10.5-15.1)	3.3	(2.2-4.9)
7-10 Years	6.0	(4.6-7.8)	6.4	(4.9-8.4)	5.0	(3.8-6.6)
11-13 Years	10.0	(8.1-12.2)	2.6	(1.9-3.6)	2.4	(1.6-3.6)
14-17 Years	19.3	(16.8-22.0)	1.1	(0.5-2.1)	1.2	(0.6-2.2)
**Socioeconomic status**						
Low	9.7	(7.2-13.0)	5.6	(3.7-8.5)	4.5	(3.0-6.9)
Medium	10.6	(9.3-12.0)	5.1	(4.2-6.1)	2.7	(2.1-3.3)
High	9.0	(7.6-10.7)	3.8	(2.9-5.1)	1.2	(0.7-2.0)
**Migration background**						
Yes	7.5	(5.7-9.8)	4.7	(3.6-6.1)	2.0	(1.2-3.2)
No	11.2	(10.0-12.5)	5.0	(4.2-5.8)	3.0	(2.4-3.7)
**Boys (total)**	**9.1**	**(8.2-10.0)**	**7.1**	**(6.4-8.0)**	**5.4**	**(4.6-6.2)**
**Age group**						
0-2 Years	11.6	(8.7-15.3)	0.6	(0.2-1.6)	0.2	(0.1-0.6)
3-6 Years	5.1	(3.9-6.8)	17.2	(14.8-19.8)	8.7	(6.7-11.2)
7-10 Years	6.4	(5.1-7.9)	10.0	(8.2-12.2)	11.4	(9.6-13.6)
11-13 Years	8.4	(6.7-10.5)	3.9	(2.9-5.3)	3.4	(2.4-4.9)
14-17 Years	14.4	(12.4-16.8)	1.4	(0.7-2.5)	1.1	(0.5-2.3)
**Socioeconomic status**						
Low	6.6	(4.9-8.9)	9.3	(7.0-12.2)	6.6	(4.8-8.9)
Medium	9.6	(8.4-11.0)	7.0	(6.1-8.0)	5.5	(4.6-6.5)
High	9.1	(7.7-10.8)	5.5	(4.5-6.7)	3.9	(3.0-5.0)
**Migration background**						
Yes	6.0	(4.5-8.0)	6.9	(5.1-9.1)	5.2	(3.6-7.3)
No	10.0	(9.0-11.2)	7.2	(6.4-8.1)	5.4	(4.7-6.2)
**Girls and Boys (total)**	**9.6**	**(8.9-10.3)**	**6.1**	**(5.5-6.6)**	**4.0**	**(3.6-4.6)**

CI=confidence interval

**Table 2:** Utilization of physical therapy, speech therapy and occupational therapy in the last 12 months among 0-to 17-year-olds by indicators of medical need (n=6,910 girls, n=6,782 boys) Source: KiGGS Wave 2 (2014-2017)

Physical therapy			Speech therapy		Occupational therapy	
%		(95 % Cl)	%	(95 % CI)	%	(95 % CI)
**Officially recognised disability (n=13,552)**						
Yes	44.7	(35.4-54.4)	31.7	(23.2-41.8)	29.9	(21.8-39.6)
No	8.9	(8.2-9.7)	5.5	(5.0-6.1)	3.6	(3.2-4.1)
**Health-related limitations (n=13,497)**						
Yes	36.8	(31.7-42.3)	19.2	(15.1-24.1)	22.2	(17.7-27.4)
No	8.4	(7.7-9.2)	5.4	(4.9-6.0)	3.3	(2.8-3.7)
**Mental health problems (participants ≥3 years) (n=12,105)**						
Normal	8.7	(8.1-9.5)	6.1	(5.4-6.8)	3.0	(2.6-3.4)
Borderline	11.4	(8.7-14.8)	10.2	(7.5-13.9)	10.5	(8.1-13.5)
Abnormal	12.0	(9.6-14.8)	14.4	(11.8-17.5)	15.9	(12.7-19.7)
**Chronic disease (n=13,539)**						
Yes	20.0	(17.2-23.2)	10.1	(8.0-12.8)	9.9	(7.8-12.4)
No	8.4	(7.8-9.2)	5.6	(5.1-6.2)	3.5	(3.0-4.0)
**Medically treated injury (n=13,579)**						
Yes	14.9	(13.2-16.9)	6.0	(4.8-7.4)	4.8	(3.6-6.4)
No	8.4	(7.7-9.2)	6.1	(5.5-6.8)	3.9	(3.4-4.3)

CI = confidence interval

**Table 3:** Sociodemografic differences in the utilization of physical therapy, speech therapy and occupational therapy over the last 12 months among 3- to 17-year-olds. Results of binary logistic regression* (n=5,937 girls, n=5,772 boys) Source: KiGGS Wave 2 (2014-2017)

Physical therapy			Speech therapy		Occupational therapy	
aOR (95 % CI)		p-value	aOR (95 % CI)	p-value	aOR (95 % CI)	p-value
**Gender**						
Boys	0.75 (0.64-0.88)	< 0.001	1.43 (1.18-1.75)	< 0.001	1.94 (1.50-2.50)	< 0.001
Girls	Ref.		Ref.		Ref.	
**Age group**						
3 – 6 Years	0.23 (0.17-0.30)	< 0.001	16.88 (9.33-28.99)	< 0.001	5.92 (3.38-10.38)	< 0.001
7 – 10 Years	0.30 (0.24-0.39)	< 0.001	7.67 (4.30-13.70)	< 0.001	7.53 (4.26-13.31)	< 0.001
11 – 13 Years	0.46 (0.36-0.58)	< 0.001	2.86 (1.63-5.00)	< 0.001	2.38 (1.28-4.44)	0.007
14 – 17 Years	Ref.		Ref.		Ref.	
**Socioeconomic status**						
Low	0.66 (0.59-0.88)	0.005	1.76 (1.27-2.25)	0.001	1.84 (1.22-2.77)	0.004
Medium	1.00 (0.84-1.19)	0.987	1.31 (1.03-1.66)	0.031	1.49 (1.09-2.03)	0.013
High	Ref.		Ref.		Ref.	
**Migration background**						
Yes	0.73 (0.58-0.93)	0.010	0.84 (0.66-1.06)	0.140	0.79 (0.54-1.13)	0.195
No	Ref.		Ref.		Ref.	

^*^all models controlled for recognised disability, mental health problems, chronic diseases as well as health-related limitations and medically treated injuries aOR=adjusted Odds Ratios, CI=confidence interval, Ref.=Reference
